# Associations of *MGMT* promoter hypermethylation with squamous intraepithelial lesion and cervical carcinoma: A meta-analysis

**DOI:** 10.1371/journal.pone.0222772

**Published:** 2019-10-01

**Authors:** Jin Huang, Jia-You Luo, Hong-Zhuan Tan

**Affiliations:** 1 Department of Epidemiology and Health Statistics, School of Public Health, Central South University, Changsha, Hunan, China; 2 Department of Women and Children Health, School of Public Health, Central South University, Changsha, Hunan, China; Academy of Sciences of the Czech Republic, CZECH REPUBLIC

## Abstract

**Background:**

In this research, an meta-analysis was performed for assessment of the associations between O^6^-methyguanine-DNA methyltransferase (*MGMT*) promoter hypermethylation possessing low-grade intraepithelial lesion (LSIL), high-grade intraepithelial lesion (HSIL), cervical cancer (CC), and clinicopathological characters of CC.

**Methods:**

Literature selection were conducted through searching PubMed, Web of science, EMBASE, China National Knowledge Infrastructure and Wanfang databases (up to November 2018). An assessment of associations between *MGMT* methylation and LSIL, HSIL, CC risk and clinicopathological characteristics was performed through pooled odds ratios (ORs) with relevant 95% confidence intervals (CIs). Subgroup analyses, meta-regressions and Galbraith plots were conducted to conduct an exploration on the possible sources of heterogeneity. The genome-wide DNA methylation array studies were extracted from Gene Expression Omnibus (GEO) databases for validation of these outcomes.

**Results:**

In this meta-analysis of 25 published articles, *MGMT* hypermethylation gradually elevated the rates among control group (12.16%), LSIL (20.92%), HSIL (36.33%) and CC (41.50%) specimens. *MGMT* promoter methylation was significant associated with the increased risk of LSIL by 1.74-fold (*P*<0.001), HSIL by 3.71-fold (*P*<0.001) and CC by 7.08-fold (*P*<0.001) compared with control. A significant association between *MGMT* promoter methylation with FIGO stage was also found (OR = 2.81, 95% CI: 1.79–4.41, *p*<0.001). The results of GEO datasets showed that 5 CpG sites in *MGMT* with a great diagnostic value for the screening of cervical cancer.

**Conclusion:**

The meta-analysis indicated the association between *MGMT* promoter hypermethylation and squamous intraepithelial lesion and cervical cancer. *MGMT* methylation detection might have a potential value to be an epigenetic marker for the clinical diagnosis of cervical cancer.

## Introduction

Cervical cancer continues to be the 2^st^ commonest gynecologic carcinoma worldwide[1], which causes approximately 528,000 new cases and 266,000 deaths per year[2]. Squamous intraepithelial lesion (SIL), a precursor of cervical cancer[3], which is featured as a progressive process from low-grade squamous intraepithelial lesion (LSIL) to high-grade squamous intraepithelial lesion (HSIL) and eventually to invasive carcinoma[4]. Human papillomavirus (HPV) infection has been widely famous to have a key function in the process of cervical carcinoma, however, merely a limited number of HPV-induced lesions eventually progress to SILs or invasive cancer[5], indicating that there were other biomolecular mechanisms in the progress of cervical carcinoma.

DNA methylation, an epigenetic modification in genes that primarily causes transcriptional silencing of genes, which played a key role in regulating transcription, embryonic development, genomic imprinting, genome stability and chromatin structure. Aberrant DNA methylation of CpG islands is comparatively seldom seen in normal cells, based on which the detection of promoter hypermethylation of tumor suppressor genes (TSGs) in bodily fluids could serve as good biomarkers for screening and prognosis of tumorigenesis development[6]. O^6^-methyguanine-DNA methyltransferase (*MGMT*) is a DNA repair enzyme removing mutagenic and cytotoxic adducts out of O^6^-guanine in DNA. Methylation of discrete regions of the CpG island of *MGMT* becomes the main cause of gene silencing and decreased expression of *MGMT* in tumor tissues and cell lines[7]. It has been proved that the gene silencing of *MGMT* is related to elevated carcinogenic risk and sensitivity to therapeutic methylating agents. Thus, the promoter methylation of *MGMT* was widely regarded as a promising biomarker for the detection of early carcinoma. Previous researches concentrated upon this in other cancers like esophageal cancer[8, 9], lung cancer[10–12], glioma[13, 14], colon cancer[15–18], gastric cancer[19], neck squamous cell carcinoma[20, 21], ovarian cancer[22] and breast cancer[23]. In 2001, *Virmani et al*.[24] first revealed an important association between the aberrant methylation of *MGMT* with the risk and histological type of cervical carcinoma. Thereafter, an increasing number of researches were conducted to investigate the association between *MGMT* hypermethylation with the process of cervical carcinogenesis. Nevertheless, the sample sizes of the studies are still small, resulting in inconsistent outcomes and a wide range of *MGMT* methylation rates in cervical carcinoma tissues. Even the opposite conclusions have also been reported in a few studies, suggesting that *MGMT* gene is rarely methylated in cervical cancer. Moreover, a meta-analysis pooled the data of 28 studies to investigate the association between *MGMT* methylation and the risk of breast and gynecologic cancers, among which 11 studies focused on cervical cancer, indicating that *MGMT* methylation and cervical cancer were positively correlated[25]. However, there were still a lack of relevant comprehensive review that systematically appraised the effect of *MGMT* promoter hypermethylation upon diverse phases of cervical carcinogenesis that from LSIL to HSIL, and finally to invasive carcinoma, as well as their clinicopathological features.

Therefore, a meta-analysis was performed for comprehensive assessment of the association of *MGMT* promoter hypermethylation with squamous intraepithelial lesion, cervical cancer and their clinicopathological characteristics.

## Methods

### Literature search

The review was reported according to the Preferred Reporting Items for Systematic Reviews and Meta-Analysis (PRISMA) 2009 guidelines (Table A in S1 File). The PubMed, EMBASE, Web of science, China National Knowledge Infrastructure (CNKI) and Wanfang databases were adopted to search candidate literature. We retrieved eligible literature updated before November 2018 by using following items “(*MGMT*) and (methylation or hypermethylation or epigene*) and (cervical cancer or cervical carcinoma or cervical tumor)”. Reference list in retrieved articles and relevant reviews were retrieved in a manual manner.

### Eligibility criteria

Eligible studies were included when satisfying the following criteria: (1) the study should be an observation designing including cohort, case-control, case-only or cross-sectional study; (2) the study should evaluated the association between *MGMT* promoter methylation and LSIL, HSIL, CC or clinicopathological characteristics of CC; (3) the study should offer enough data for calculation of odds ratios (ORs) and 95% confidence intervals (CIs); (4) the study should be written in English or Chinese.

Exclusion criteria were as follows: (1) they were meeting abstracts, reviews, letters or case reports; (2) they regarding in *vitro* or *ex vivo* experiments of cell lines or animals.

### Data extraction

The data of eligible studies were extracted by two independent authors. The following information of each eligible study were collected: the first author’s name, publication year, ethnicity, country, study design, sample size, methylation detection methods, materials, source of controls, involved diseases (LSIL, HSIL, CC), their clinicopathological characteristics (age at diagnose, HPV infection, histological type, FIGO stage, therapeutic response, histological grade, lymph node metastasis) and quality of studies.

### Validation by GEO datasets

The genome-wide DNA methylation array studies were extracted from Gene Expression Omnibus (GEO) databases by using following items “Cervical cancer”, “Methylation” and “Homo sapiens”. Eligible criteria were as follows: (1) the quantitative methylation levels of datasets were detected by the Illumina HumanMethylation 27 or 450 k Beadchip; (2) datasets using cohort or case-control designs. Exclusion criteria were as follows: (1) datasets regarding in *vitro* or *ex vivo* experiments of cell lines or animals; (2) datasets without CpG number.

The following information of each eligible datasets were collected: submission and last update date, ethnicity, country, sample size, methylation detection methods, source of controls, involved diseases (LSIL, HSIL, CC).

### Quality assessment of eligible studies and datasets

The quality of eligible studies and datasets was assessed by two independent authors (JH and JYL) in line with a preset system derived from the REMARK [26] and BRISQ [27] guidelines. 18 items were considered as quality components, including study design, study population, biospecimen information, methylation detection, clinicopathological characteristics and outcomes analysis (Table B in S1 File). Studies reporting exceeding 11 items were considered to be high-quality studies.

### Statistical methods

The *MGMT* promoter hypermathylation rates in LSIL, HSIL and CC specimens were calculated by the inverse variance approach [28]. Cochran-Armitage (CA) trend test were used to compare the methylation frequency in control group, LSIL, HSIL and CC specimens. Pooled ORs and their 95% CIs were calculated to estimate the association between *MGMT* promoter methylation possessing LSIL, HSIL, CC and their clinicopathological characteristics. Heterogeneity across the included studies were assessed by the Cochran’s Q test and *I*^2^ statistic. *I*^2^ value of greater than 25%, 50% and 75% meant mild, moderate and high heterogeneity, respectively[29]. When significant heterogeneity (*I*^2^ value larger than 50% or *P*_Q-test_ smaller than 0.1) was observed, the random-effect model was utilized to pool the results, otherwise, a fixed-effect was applied[30]. To further explore the potential source of heterogeneity, subgroup analyses and meta-regression were performed based on ethnicity, source of controls, materials, published year (≥2010 and <2010) and quality of studies. And, Galbraith plots were further depicted to seek the impact of individual studies of the overall heterogeneity. Moreover, sensitivity analysis was conducted through sequentially removing every study or heterogeneity spotted by Galbraith plots to further assess the stability of the pooled outcomes. In GEO datasets, the relationship between CpG sites of *MGMT* and cervical cancer were calculated by Mann-Whitney U test. Trial sequential analysis (TSA) was performed by TSA 0.9 software (Copenhagen Trial Unit, Center for Clinical Intervention Research, Denmark, http://www.ctu.dk/tsa/) with type I errors of 5%, type II errors of 20% and a statistical test power of 80%. Publication bias was evaluated by funnel plots and Egger’s test[31]. Funnel plot and *P*_Egger_≤0.05 indicated the presence of publication bias. All the statistical analysis above were undertaken by RevMan 5.3 (The Nordic Cochrane Centre, The Cochrane Collaboration) and Stata 15.0 (Stata, College, TX, USA).

## Results

### Characteristics of included studies

**Fig 1:**
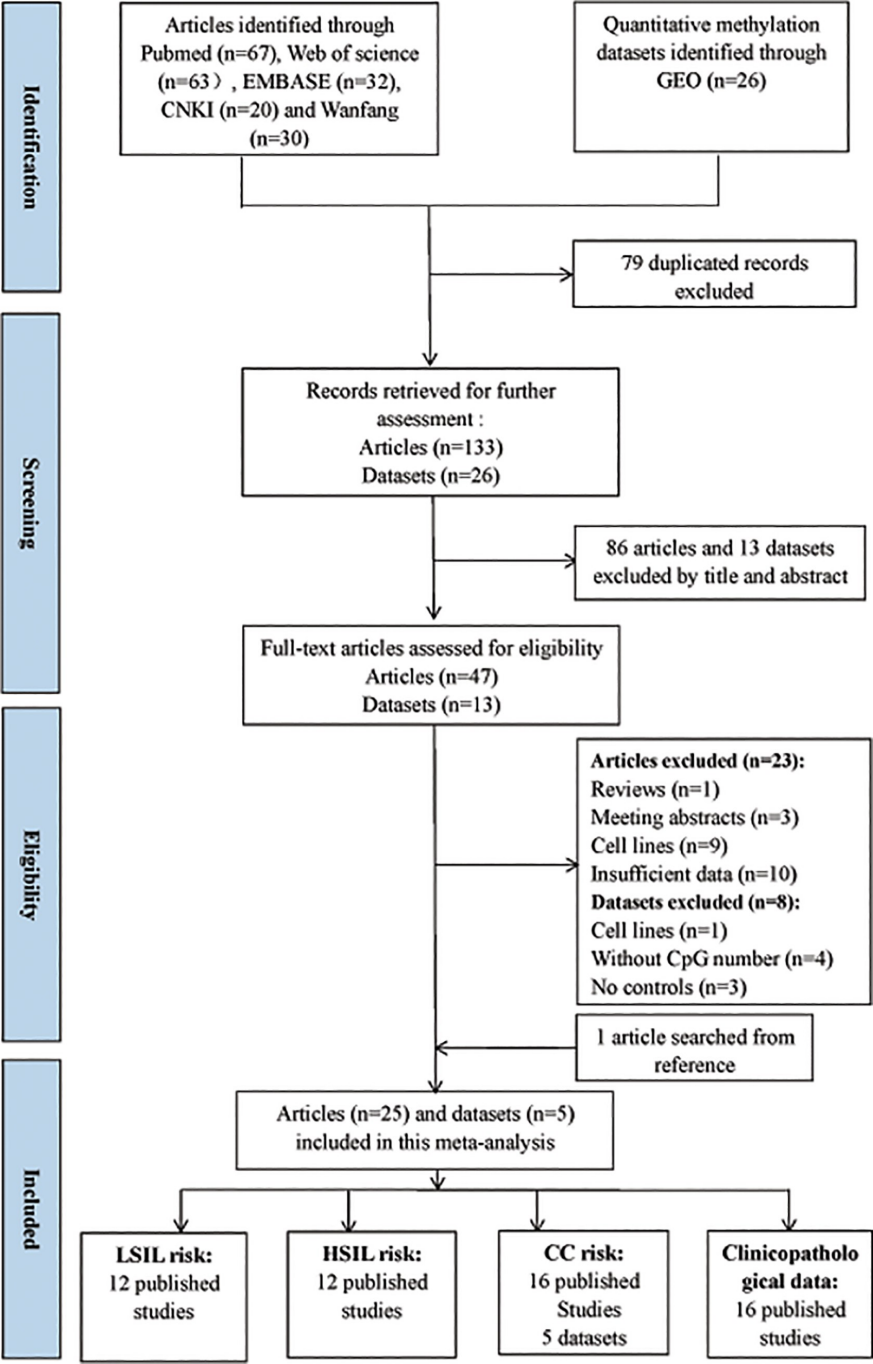
Flow diagram for the procedures of eligible studies selection in this meta-analysis.

Based on the definitions of the 2001 Bethesda System[32], the category of LSIL encompassed cytopathic effects of HPV, cervical intraepithelial neoplasia (CIN) 1 and mild dysplasia. The category of HSIL contained moderate or extensive dysplasia and CIN 2 or 3. CC contained squamous cell carcinoma (SCC) and adenocarcinoma (AdC). Upon the basis of such definitions and the literature search, 47 full-text articles were initially selected and assessed for eligibility. Then, 23 articles were excluded because of reviews (n = 1), meeting abstracts (n = 3), cell lines (n = 9) and insufficient data (n = 10). Manual search of reference cited in the published articles spotted one additional study[33]. Finally, a total of 25 articles [33–55] were included in this meta-analysis. Of such studies, all studies were eligible to calculate the hypermethylation rates of *MGMT*. A total of 2933 patients with SIL or CC from 25 studies (6 case-only studies[34, 43, 46, 48, 49, 51] and 19 case-control studies) were eligible to estimate the association of *MGMT* methylation status with the clinicopathological features. For most of these 25 studies (17 of 25), the detection of *MGMT* promoter methylation was performed by methylation-specific PCR (MSP). Besides, two studies performed by HRM, only one study performed by pyrosequencing[46], one study performed by MS-MLPA[48], two studies performed by QMSP[45, 53], as well as two studies performed by MSP and sequencing[51, 55]. Among these 25 studies, 11 studies used exfoliated cells of cervical samples to detect *MGMT* methylation status, while other 10 studies involved cervical tissues, 3 studies involved tissue and plasma and one study only involved plasma. Regarding the type of ethnicity, eighteen studies were carries out on Asian, seven studies on Caucasians. The flow diagram for the process of included articles in this meta-analysis was revealed in Fig 1. The detailed characteristics of included articles were listed in Table 1.

**Table 1:** Characteristics of included studies in this meta-analysis.

No.	Author	Year	Country	Ethnicity	Study design	Sample size				Methylation detection method	Materials	Source of controls	Involved clincopathological features	Quality scores
						Control	CC	HSIL	LSIL					
1	Lodi	2018	Germany	Caucasian	Case-only	-	143	-	-	MSP	Tissue	-	Therapeutic response	10
2	L.-L Sun	2018	China	Asian	Case-control	45	5	-	-	MS-HRM	Exfoliated cells	B	-	11
3	Wang^a^	2018	China	Asian	Case-control	138	-	98	107	MSP	Exfoliated cells	B	-	10
4	Nan^a^	2016	China	Asian	Case-control	156	-	134	147	MSP	Exfoliated cells	H	-	11
5	Yin Sun	2015	China	Asian	Case-control	48	45	103	54	MS-HRM	Exfoliated cells	B	-	10
6	Banzai	2014	Japan	Asian	Case-control	24	53	-	-	MSP	Tissue	B	Histological type	10
7	Lu^a^	2014	China	Asian	Case-control	20	50	100	50	MSP	Exfoliated cells	B	FIGO stage,Histological grade	11
8	Sun	2012	China	Asian	Case-control	336	-	37	68	MSP	Exfoliated cells	H	Age,HPV	14
9	Jian^a^	2012	China	Asian	Case-control	30	52	-	-	MSP	Tissue	B	FIGO stage, Histological grade	11
10	Spathis	2011	Greece	Caucasian	Case-control	57	18	101	164	MSP	Exfoliated cells	H	Histological type	12
11	Liu^a^	2011	China	Asian	Case-only	-	183	-	-	MSP	Plasma	-	HPV,histological type	12
12	Kim	2010	Korea	Asian	Case-control	41	69	67	32	MSP	Exfoliated cells	B	-	12
13	Iliopoulos	2009	Greece	Caucasian	Case-control	27	61	12	15	QMSP	Exfoliated cells	H	FIGO stage	12
14	Flatley	2009	UK	Caucasian	Case-control	45	42	102	49	MSP	Exfoliated cells	H	-	10
15	Lee	2008	Korea	Asian	Case-only	-	34	-	-	Pyro- sequencing	Tissue	-	Histological type, FIGO stage,lymph node metastasis	10
16	Chen^a^	2008	China	Asian	Case-control	38	86	-	-	MSP	Tissue and plasma	H	FIGO stage, histological grade,lymph node metastasis	12
17	Henken	2007	Nether- lands	Caucasian	Case-only	-	29	-	-	MS-MLPA	Tissue	-	Histological type	11
18	Hoenil	2007	Korea	Asian	Case-only	-	82	-	-	MSP	Tissue	-	-	10
19	Gao^a^	2007	China	Asian	Case-control	15	38	15	5	MSP	Tissue	B	Histological grade,lymph node metastasis, FIGO stage	10
20	Yang	2006	China	Asian	Case-only	-	127	-	-	MSP and sequencing	Tissue	-	FIGO stage, histological grade, histological type, therapeutic response	13
21	Lin	2005	Korea	Asian	Case-control	20	67	20	10	MSP	Tissue	H	Histological type	11
22	Reesink-Peters	2004	Netherlands	Caucasian	Case-control	41	48	-	-	QMSP	Exfoliated cells	B	-	10
23	Yang	2004	China	Asian	Case-control	100	85	-	-	MSP	Tissue and plasma	A	Histological type,FIGO stage, histological grade	13
24	Dong	2001	Korea	Asian	Case-control	24	53	-	-	MSP and sequencing	Tissue	B	Histological type,FIGO stage, histological grade,age	13
25	Virmani	2001	USA	Caucasian	Case-control	22	19	17	37	MSP	Tisses,blood lymphocytes and buccal epithelial	H	-	13

**Abbreviations:** CC, cervical cancer; LSIL, low-grade squamous intra-epithelial lesion; HSIL, high-grade squamous intra-epithelial lesion; MSP, methylation-specific PCR; H, healthy controls; B, controls with benign gynecological diseases; A, autologous controls.

Notes

^a^ Studies written in Chinese

### Pooled rates of *MGMT* hypermethylation in patients with LSIL, HSIL and CC

Altogether 1227 controls, 738 LSIL, 827 HSIL and 791 CC specimens were included in this meta-analysis. As shown in Table 2, the pooled rates of *MGMT* hypermethylation demonstrated a progressively increased trend (*p*<0.001) from control group (12.16%, 95% CI: 4.43–20.81%) to LSIL (20.92%, 95% CI: 9.22–32.62%), to HSIL (36.33%, 95% CI: 24.95–47.72%) and eventually to CC (41.50%, 95% CI: 28.19–54.81%) specimens.

**Table 2:** Pooled hypermethylation rates of *MGMT* in LSIL, HSIL and CC specimens.

Comparisons	Studies	Specimens	Methylation rates (%)	95% CI (%)
Control	19	1227	12.16%	4.43–20.81
LSIL	12	738	20.92%	9.22–32.62
HSIL	12	828	36.33%	24.95–47.72
CC	16	791	41.50%	28.19–54.81

### The relationship between *MGMT* promoter hypermethylation and LSIL risk

**Fig 2:**
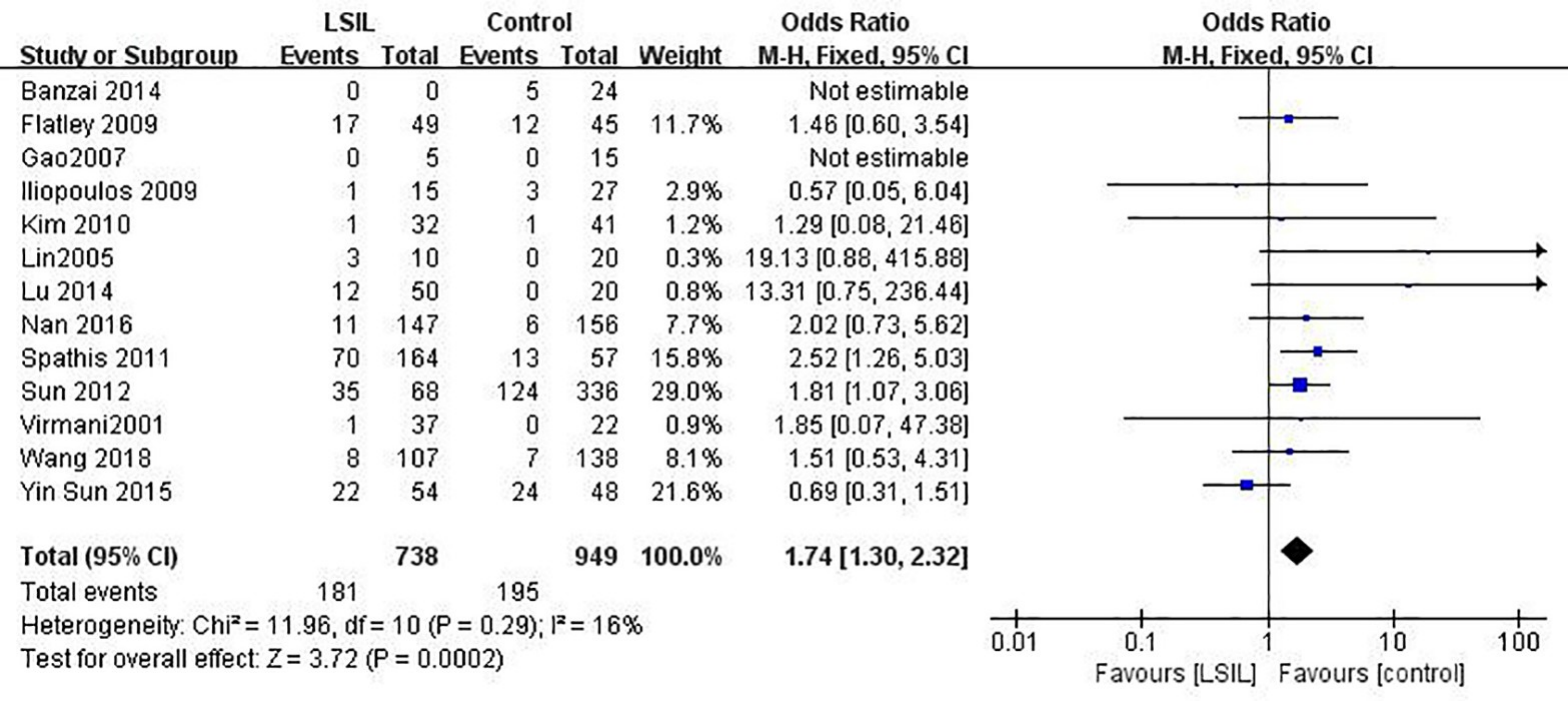
Funnel plots for associations of *MGMT* promoter hypermethylation with the risk of LSIL. The squares represent the ORs for individual studies. The size of the square reflects the weight of included studies. Bars represent the 95% confidence intervals (CIs). The center of the diamond represents the summary effect size. LSIL, low-grade intra-epithelial lesion.

Twelve published studies including 738 patients with LSIL risk and 925 controls were included to estimate the effect of *MGMT* promoter hypermethylation on LSIL risk (Fig 2). *MGMT* promoter hypermethylation conferred a 1.75-fold (95% CI: 1.30–2.32) elevated risk of LSIL and a *p* value of <0.001 (Table 3). In ethnicity based subgroup analysis, there were no significant differences in methylation rates between Asians (OR: 1.65, 95% CI: 1.16–2.35) and Caucasians (OR: 1.93, 95% CI: 1.15–3.22). Such association was still significant in most subgroups except for the “non-healthy”, “tissue”, “publication year before 2010” and “Quality of studies lower than 11”. There was no significant heterogeneity in all comparisons (*I*^2^: 0–39%).

**Table 3:** Pooled results for the association of *MGMT* promoter hypermethylation with LSIL risk.

Comparisons	Studies (N)	Sample size (LSIL/controls)	Heterogeneity		Model^a^	Effect size	
			I^2^(%)	*P*_Q-text_		OR (95% CI)	*P*
Total	12	738/925	16	0.29	F	1.74(1.30–2.32)	<0.001
Ethnicity							
Asian	8	473/774	37	0.14	F	1.65(1.16–2.35)	0.005
Caucasian	4	265/151	0	0.58	F	1.93(1.15–3.22)	0.010
Source of controls							
Healthy	7	490/663	0	0.65	F	1.97(1.40–2.78)	<0.001
Non-healthy^b^	5	248/262	39	0.18	F	1.23(0.70–2.15)	0.480
Materials							
Tissue	3	52/57	5	0.31	F	6.71(0.78–57.61)	0.080
Exfoliated cells	9	686/868	16	0.30	F	1.68(1.25–2.25)	<0.001
Publication year							
≥ 2010	7	622/796	31	0.19	F	1.75(1.27–2.40)	<0.001
< 2010	5	116/129	9	0.35	F	1.70(0.82–3.53)	0.160
Quality of studies							
High (>11)	7	376/523	0	0.48	F	2.24(1.52–3.29)	<0.001
Low (≤11)	5	362/402	10	0.35	F	1.22(0.78–1.91)	0.390

**Abbreviations:** N, number; LSIL, low squamous intra-epithelial lesion; F, fixed-effects model; R, random-effects model.

Notes

^a^ When significant heterogeneity was found (*I*^2^≥50% or *P*_Q-test_≤0.1), a random-effects model with the inverse variance method was used to pool the results; otherwise, a fixed-effects model was applied.

^b^ Non-healthy controls included autologous controls and controls with benign gynecological diseases.

### The relationship between *MGMT* promoter hypermethylation and HSIL risk

**Fig 3:**
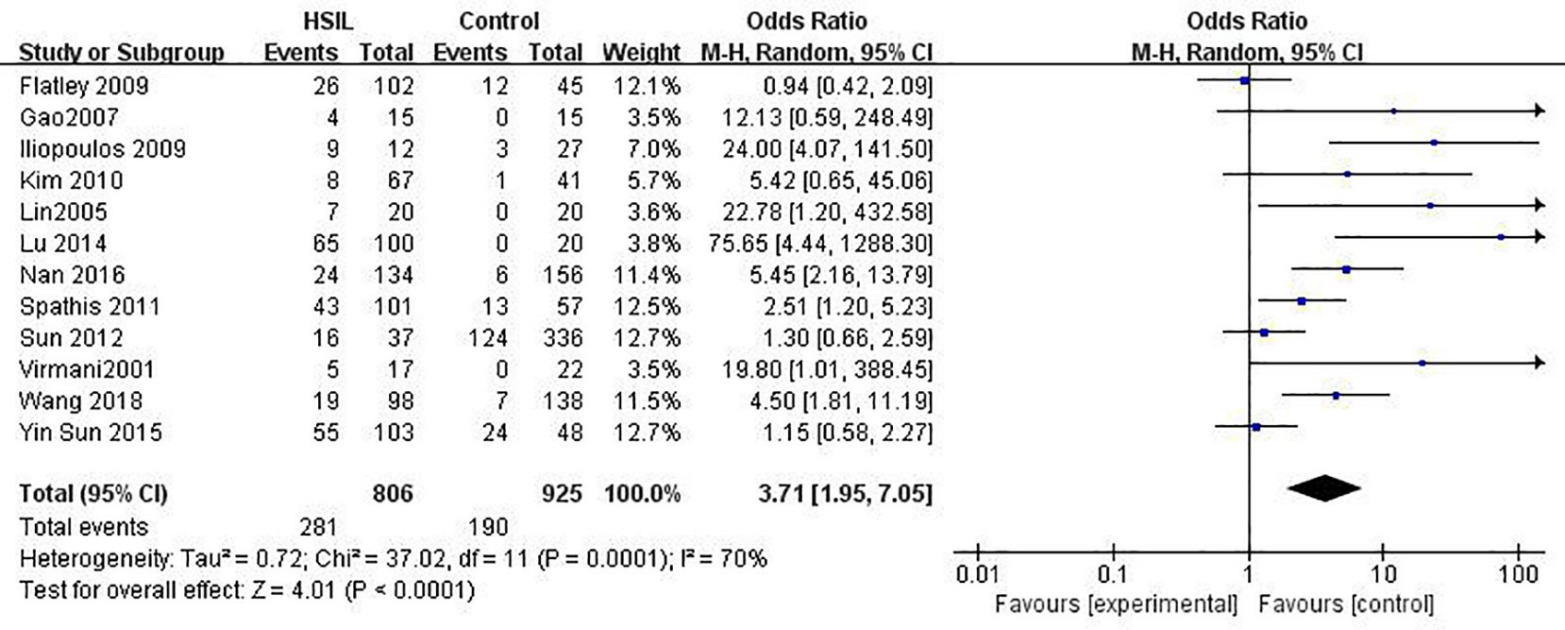
Funnel plots for associations of *MGMT* promoter hypermethylation with the risk of HSIL. The squares represent the ORs for individual studies. The size of the square reflects the weight of included studies. Bars represent the 95% confidence intervals (CIs). The center of the diamond represents the summary effect size. HSIL, high-grade intra-epithelial lesion.

A total of 828 patients with HSIL and 949 controls from 12 studies were eligible to assess the association of *MGMT* promoter methylation status with HSIL risk (Fig 3). Overall, *MGMT* promoter hypermethylation was associated with a 3.37-fold (95% CI: 1.86–6.14) increased risk of HSIL and a *p* value of <0.001 (Table 4). The association was still significant in all subgroups as shown in Table 3.

**Table 4:** Pooled results for the association of *MGMT* promoter hypermethylation with HSIL risk.

Comparisons	Studies (N)	Sample size (HSIL/controls)	Heterogeneity		Model^a^	Effect size	
			I^2^(%)	*P*_Q-text_		OR (95% CI)	*P*
Total	12	806/925	70	<0.001	R	3.71(1.95–7.05)	<0.001
Ethnicity							
Asian	8	574/774	70	0.001	R	3.93(1.73–8.90)	0.001
Caucasian	4	232/151	78	0.003	R	3.89(1.05–14.35)	0.040
Source of controls							
Healthy	7	423/663	73	0.001	R	3.46(1.52–7.86)	0.003
Non-healthy^b^	5	383/262	74	0.004	R	4.95(1.36–17.98)	0.010
Materials							
Tissue	3	74/81	0	0.950	F	17.97(3.23–9.90)	0.001
Exfoliated cells	9	754/868	74	<0.001	R	3.04(1.57–5.87)	<0.001
Publication year							
≥ 2010	7	640/796	69	0.003	R	2.94(1.50–5.74)	0.002
< 2010	5	166/129	77	0.002	R	8.57(1.28–57.53)	0.030
Quality of studies							
High (>11)	7	234/483	67	0.020	R	4.00(1.46–10.92)	0.007
Low (≤11)	5	572/442	76	<0.001	R	3.84(1.49–9.93)	0.005

**Abbreviations:** N, number; HSIL, high squamous intra-epithelial lesion; F, fixed-effects model; R, random-effects model.

Notes

^a^When significant heterogeneity was found (*I*^2^≥50% or *P*_Q-test_≤0.1), a random-effects model with the inverse variance method was used to pool the results; otherwise, a fixed-effects model was applied.

^b^ Non-healthy controls included autologous controls and controls with benign gynecological diseases.

Due to observation of moderate heterogeneity in the overall comparison (*I*^2^ = 70%), subgroup, meta-regression and Galbraith plot analyses were performed to explore the potential sources of heterogeneity. In ethnicity based subgroup analysis, there were no significant differences in methylation rates between Asians (OR: 3.93, 95% CI: 1.73–8.90) and Caucasians (OR: 3.89, 95%CI: 1.05–14.35). Moderate heterogeneity remained in most of the subgroups, except for the “tissues” subgroup (*I*^2^ = 0%). The outcomes of meta-regression analyses illustrated that ethnicity (*p* = 0.978), source of controls (*p* = 0.999), materials (*p* = 0.513), publication year (*p* = 0.340) and quality of studies (*p* = 0.752) were all not main sources of heterogeneity (Table C in S1 File). Furthermore, a Galbraith plot was further depicted, spotting four outliers [33, 42, 56, 57] as major sources of heterogeneity (Figure A in S1 File). Such four studies were all classified into “exfoliated cells” studies, and exclusion of such four studies caused a decline in *I*^2^ value from 68 to 4%, followed by an apparent association between the methylation of *MGMT* with increased HSIL risk (OR = 8.51, 95% CI: 5.02–14.42, *p*<0.001).

### The relationship between *MGMT* promoter hypermethylation and CC risk

**Fig 4:**
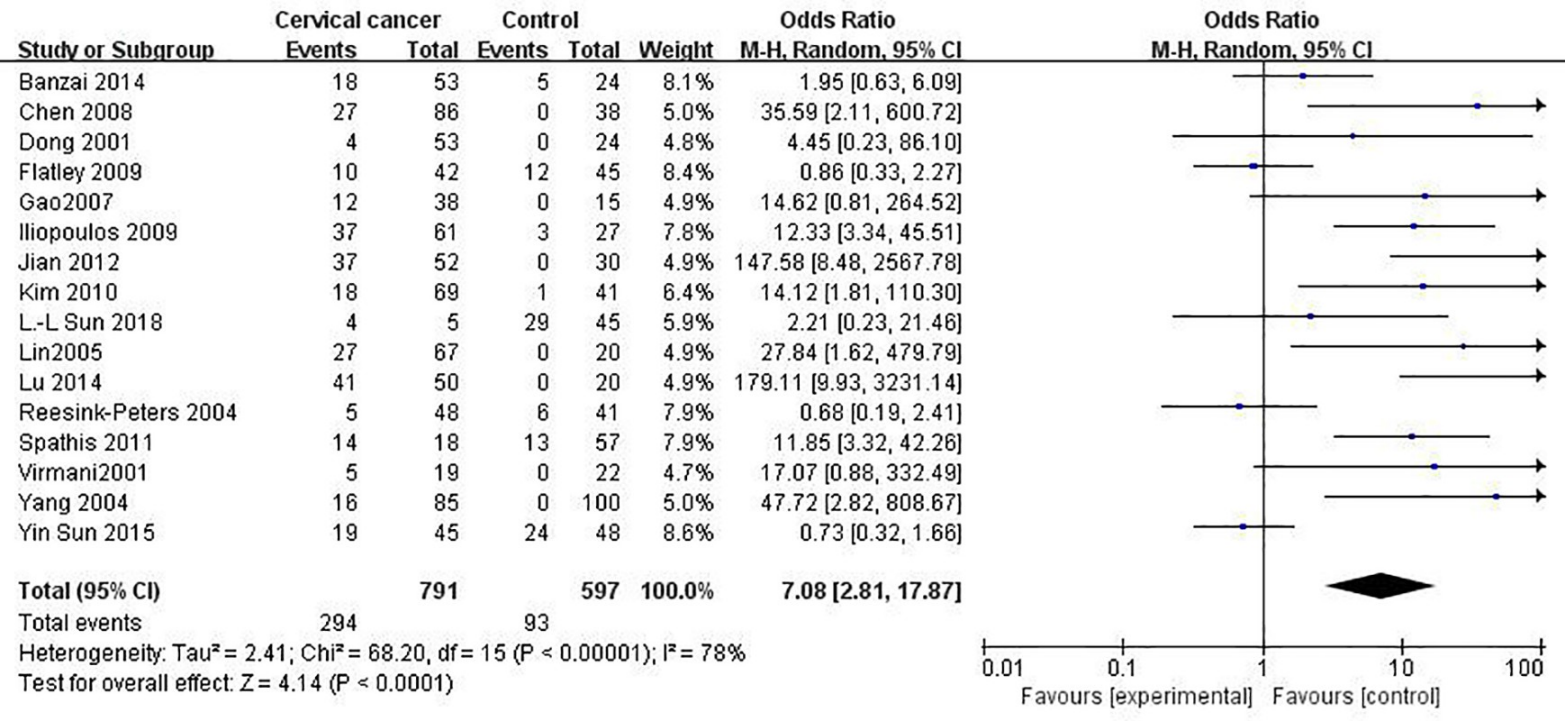
Funnel plots for associations of *MGMT* promoter hypermethylation with the risk of cervical cancer. The squares represent the ORs for individual studies. The size of the square reflects the weight of included studies. Bars represent the 95% confidence intervals (CIs). The center of the diamond represents the summary effect size.

16 published studies including 791 CC patients and 597 controls were included to assess the effect of *MGMT* promoter hypermethylation upon CC risk (Fig 4). *MGMT* promoter hypermethylation conferred a 7.08-fold (95% CI: 2.81–17.87) increased risk of CC and a *p* value of <0.001 (Table 5).

**Table 5:** Pooled results for the association of *MGMT* promoter hypermethylation with CC.

Comparisons	Studies (N)	Sample size (CC/controls)	Heterogeneity		Model^a^	Effect size	
			I^2^(%)	*P*_Q-text_		OR (95% CI)	*P*
Total	16	791/597	78	<0.001	R	7.08(2.81–17.87)	<0.001
Ethnicity							
Asian	11	603/405	79	<0.001	R	11.12(2.95–41.95)	<0.001
Caucasian	5	188/192	82	<0.001	R	3.67(0.87–15.46)	0.08
Source of controls							
Healthy	6	293/209	76	<0.001	R	8.92(2.14–37.28)	0.003
Non-healthy^b^	10	498/388	80	<0.001	R	6.41(1.77–23.27)	0.005
Materials							
Tissue	8	453/273	55	0.03	R	15.38(4.06–58.30)	<0.001
Exfoliated cells	8	338/324	83	<0.001	R	3.87(1.18–12.67)	0.03
Publication year							
≥ 2010	7	292/265	84	<0.001	R	7.84(1.76–34.93)	0.007
< 2010	9	499/	74	<0.001	R	6.88(1.89–25.00)	0.003
Quality of studies							
High (>11)	10	560/379	0	0.59	F	23.96(12.41–46.20)	<0.001
Low (≤11)	6	231/218	21	0.27	F	1.16(0.74–1.84)	0.52

**Abbreviations:** N, number; CC, cervical cancer; F, fixed-effects model; R, random-effects model.

Notes

^a^When significant heterogeneity was found (*I*^2^≥50% or *P*_Q-test_≤0.1), a random-effects model with the inverse variance method was used to pool the results; otherwise, a fixed-effects model was applied.

^b^ Non-healthy controls included autologous controls and controls with benign gynecological diseases.

Due to observation of extensive heterogeneity was observed in the overall comparison (*I*^2^ = 78%), subgroup, meta-regression and Galbraith plot analyses were conducted for seeking the possible sources of heterogeneity. In subgroup analyses, such association was till significant in nearly every subgroups in addition to the low-quality studies. However, moderate or extensive heterogeneity was still in most of the subgroups, except for the subgroups involving high-quality studies (*I*^2^ = 0%). The outcomes of meta-regression analyses illustrated that ethnicity (*p* = 0.248), source of controls (*p* = 0.880), materials (*p* = 0.654) and publication year (*p* = 0.570) were not main sources of heterogeneity (Table D in S1 File). Only the quality of studies was the major source of heterogeneity (*p*<0.001). Moreover, a Galbraith plot was further depicted, spotting four outliers [33, 37, 53, 56] as major sources of heterogeneity (Figure B in S1 File). These four studies were all classified into low-quality studies, and exclusion of such four studies caused a decline in *I*^2^ value from 78 to 0%, followed by a significant association between the methylation of *MGMT* with increased CC risk (OR = 20.31, 95% CI: 11.02–37.41, *p*<0.001), which further providing support to the outcomes of meta- regression.

### The relationship between *MGMT* promoter hypermethylation and clinicopathological feature of cervical cancer

**Fig 5:**
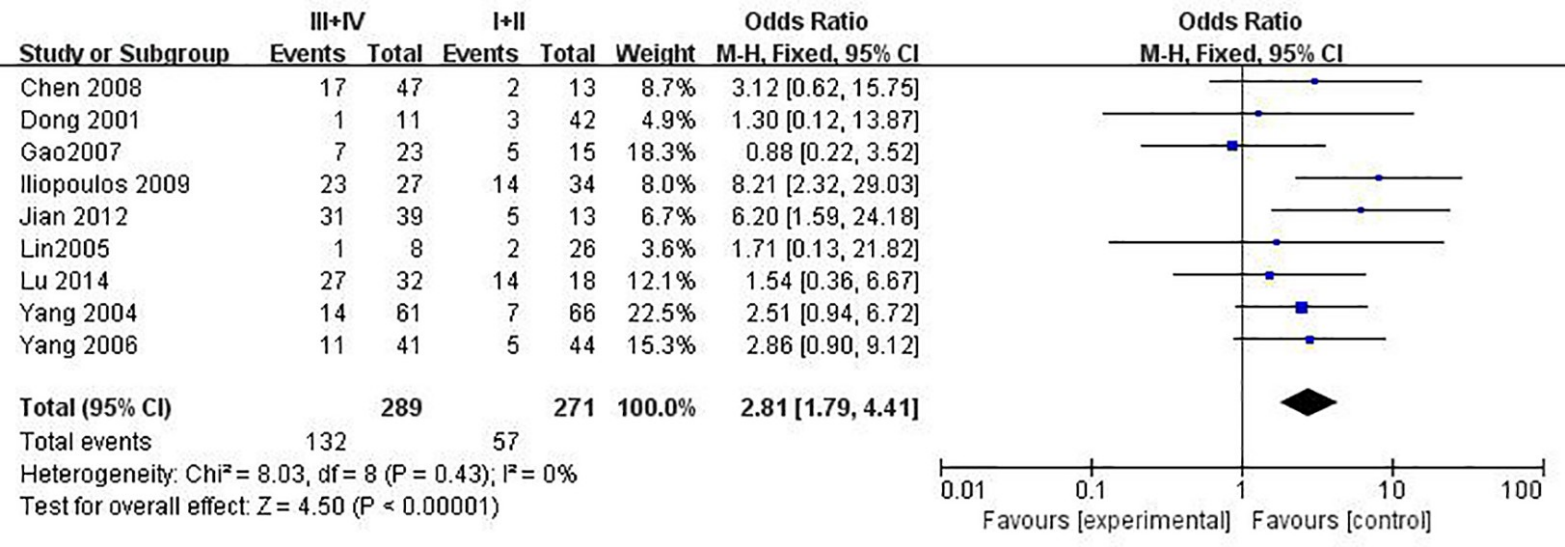
Funnel plots for associations of *MGMT* promoter hypermethylation with the FIGO stage of cervical cancer. The squares represent the ORs for individual studies. The size of the square reflects the weight of included studies. Bars represent the 95% confidence intervals (CIs). The center of the diamond represents the summary effect size.

**Fig 6:**
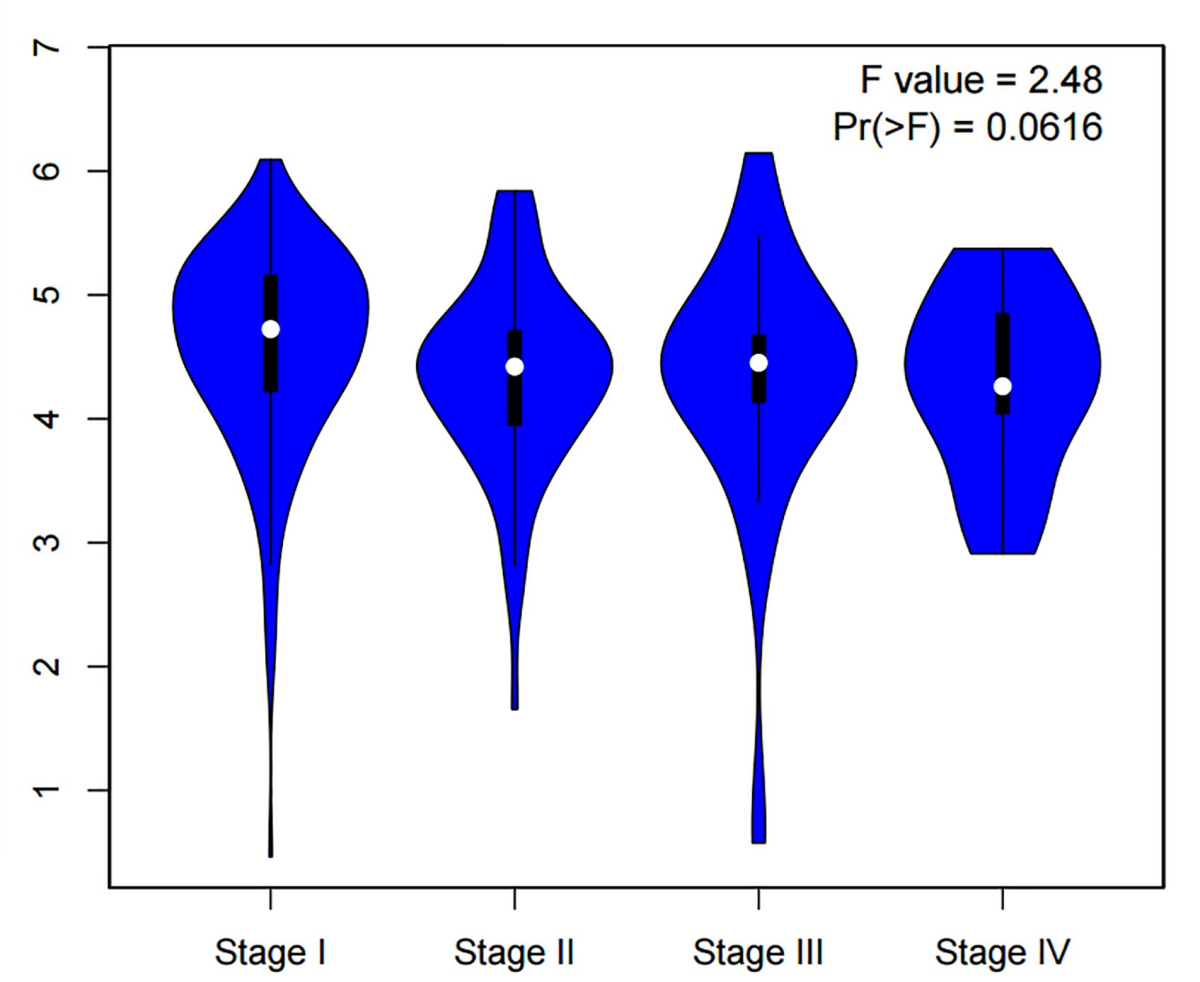
The levels of *MGMT* expression in different FIGO stage of cervical cancer (*p*>0.05) from GEPIA databases.

Through combination of the methylation data from 16 published studies including 1676 SIL or CC patients, we evaluated the relationship between *MGMT* promoter methylation and clinicopathological features including histological types, advanced International Federation of Gynecology and Obstetrics (FIGO) stage, histological grade, HPV infection, therapeutic response, age at diagnoses and lymph node metastasis. As shown in Table 6 and Fig 5, *MGMT* promoter methylation was significantly associated with FIGO stage (OR = 2.81, 95% CI:1.79–4.41, *p*<0.001), but not with histological types (see Figure C in S1 File), histological grade (see Figure D in S1 File), HPV infection, therapeutic response, age and lymph node metastasis. Furthermore, the results from the Gene Expression Profiling Interactive Analysis (GEPIA) databases demonstrated that there were no significant association between *MGMT* expression and FIGO stage (*p*>0.05) (Fig 6).

**Table 6:** Poole results for the associations between *MGMT* promoter hypermethylation and clincopathological features of CC.

Clincopathological features	Studies (N)	Patients (N)	Heterogeneity		Model^a^	Effect size	
			I^2^(%)	*P*_Q-test_		OR (95% CI)	P
Histological types (SCC vs. AdC)	8	475	0	0.73	F	0.73(0.43–1.26)	0.26
FIGO stage (I+II vs. III+IV)	9	560	0	0.43	F	2.81(1.79–4.41)	**<0.001**
Histological grade(G3 vs. G1+G2)	7	433	54	0.04	R	1.15(0.49–2.68)	0.74
HPV infection(Positive vs. Negative)	2	850	96	<0.001	R	17.24(0.02–190.55)	0.43
Therapeutic response (Yes vs. No)	2	206	0	0.85	F	1.65(0.80–3.38)	0.17
Age at diagnoses (<50 vs. ≥50)	2	720	0	0.98	F	1.40(0.93–2.10)	0.11
Lymph node metastasis (Yes vs. No)	2	66	1	0.32	R	4.91(1.56–15.42)	0.007

**Abbreviations:** N, number; SCC, squamous cell carcinoma; AdC, adenocinoma; F, fixed-effects model; R, random-effects model.

Notes

^a^When significant heterogeneity was found (I^2^≥50% or *P*_Q-test_≤0.1), a random-effects model with the inverse variance method was used to pool the results; otherwise, a fixed-effects model was applied.

### Prognostic role of *MGMT* expression in cervical cancer

**Fig 7:**
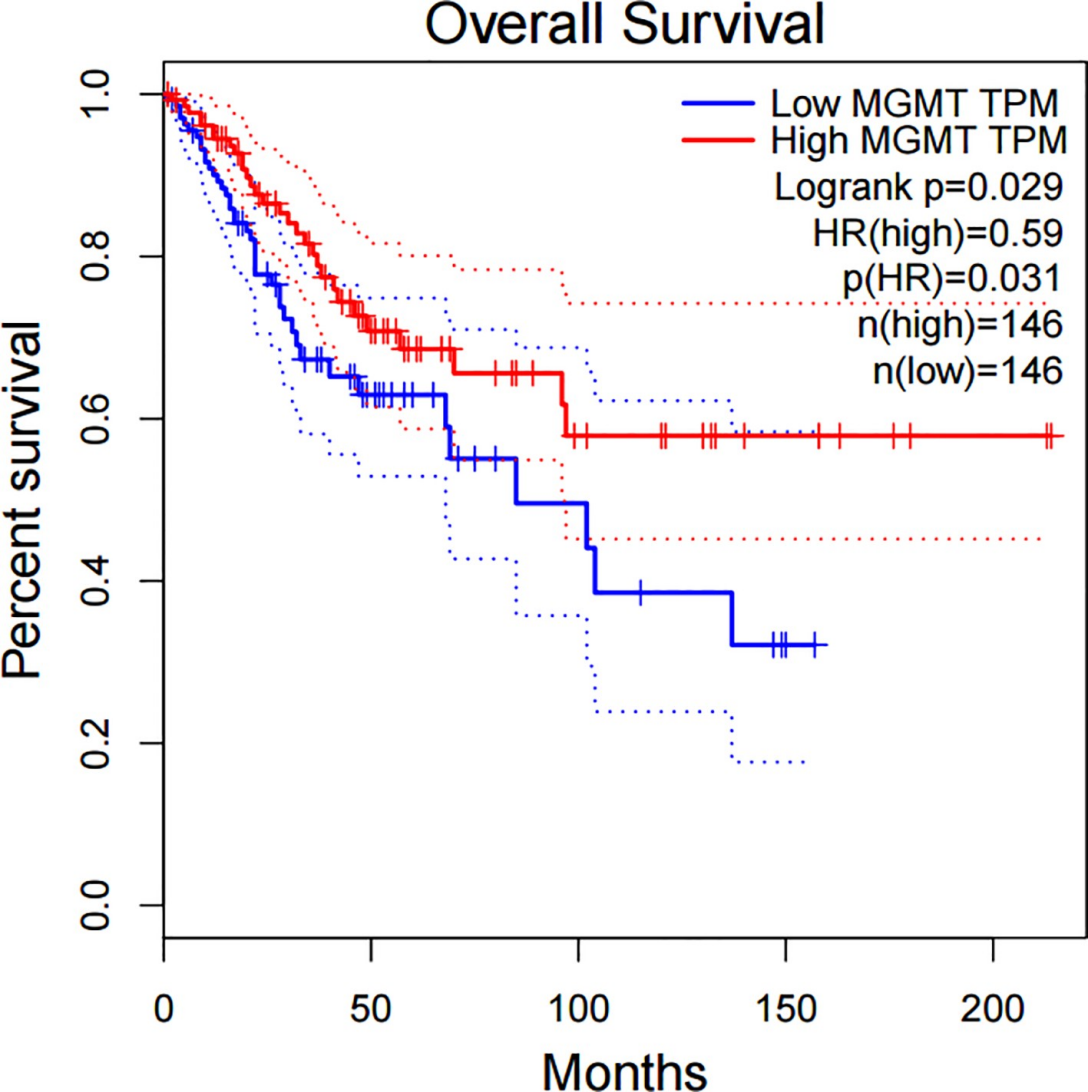
The correlation between *MGMT* expression and overall survival in cervical cancer (*p*<0.05) from GEPIA databases.

The further analysis from GEPIA databases illustrated that *MGMT* expression was related to overall survival (OS) in 292 patients with cervical cancer (*p*<0.05), as shown in Fig 7.

### Validation by quantitative methylation data from GEO databases

**Fig 8:**
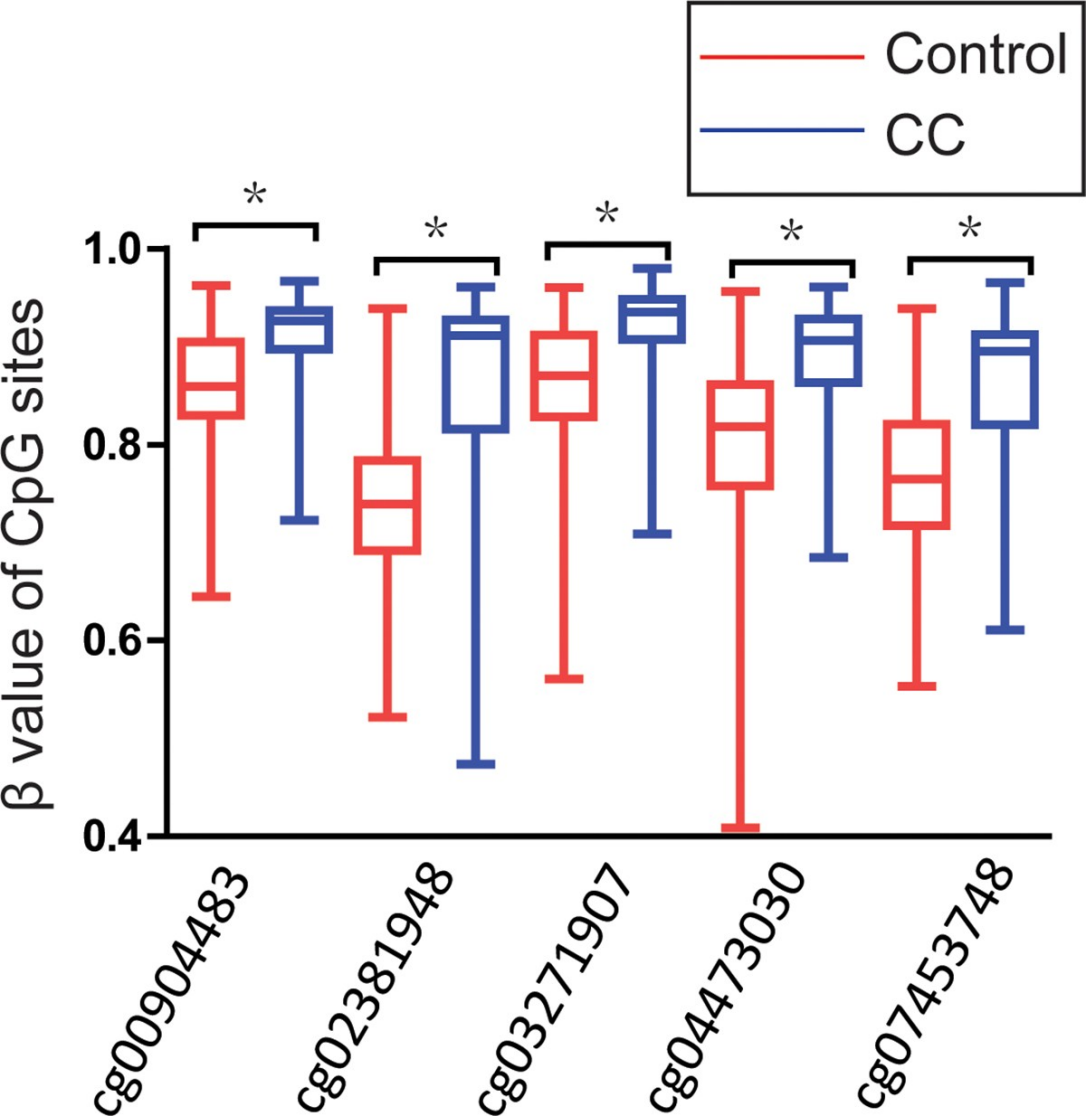
Significant differences of methylation level in 5 CpG sites of *MGMT* between cervical cancer and controls in GEO dataset. *P*-value were calculated by the Mann-Whitney U test. *P<0.001.

The genome-wide DNA methylation array studies were extracted from GEO databases to validate the results. Totally, 5 datasets (GSE99511, GSE46306, GSE41384, GSE36637 and GSE30760) involved genome-wide DNA methylation array of 67 controls, 63 patients with cervical cancer, as shown in Fig 7. A total of 7 CpG sites (cg00904483, cg02381948, cg02803836, cg02941816, cg03271907, cg04473030, cg07453748) in promoter region of *MGMT* were included. 5 of 7 CpG sites (cg00904483, cg02381948, cg03271907, cg04473030, cg07453748) showed significance results with *p*-values<0.001 when methylation level of cervical cancer compared with that of controls (as shown in Fig 8, Table 7). Besides, these 5 CpG sites showed a great diagnostic value for cervical cancer with AUC from 0.779 to 0.818, specificities from 0.682 to 0.848, sensitivities from 0.700 to 0.829, which were shown in bold in Table 8.

**Table 7:** Diagnostic value of 7 CpG sites of *MGMT* promoter for cervical cancer.

CpG sites	CpG island	CpG island location	Diagnostic value of CpG sites in CC				*p*-value
			Cut-off value	Specificity	Sensitivity	AUC	
**cg00904483**	**TRUE**	**chr10:131244704–131245359**	**0.878**	**0.682**	**0.829**	**0.776**	**3.39E-07**
**cg02381948**	**TRUE**	**chr10:131244704–131245359**	**0.795**	**0.788**	**0.814**	**0.817**	**1.33E-10**
cg02803836	TRUE	chr10:131302456–131302963	0.914	0.606	0.657	0.548	0.390
cg02941816	TRUE	chr10:131154808–131155770	0.053	0.591	0.714	0.606	0.295
**cg03271907**	**TRUE**	**chr10:131302456–131302963**	**0.900**	**0.742**	**0.800**	**0.802**	**7.96E-08**
**cg04473030**	**TRUE**	**chr10:131244704–131245359**	**0.879**	**0.833**	**0.714**	**0.818**	**5.99E-09**
**cg07453748**	**TRUE**	**chr10:131244704–131245359**	**0.862**	**0.848**	**0.700**	**0.797**	**4.26E-09**

**Abbreviations:** CC, cervical cancer; AUC, area under the curve.

**Table 8:** Characteristics of included GEO datasets in this meta-analysis.

Author	Year	Country	Ethnicity	Sample size		Methylation detection method	Materials	Source of controls	Quality scores
				Control	CC				
GSE99511	2017–2019	Netherlands	Caucasian	28	4	Illumina HumanMethylation450 BeadChip		B	11
GSE46306	2013–2019	Sweden	Caucasian	20	6	Illumina HumanMethylation450 BeadChip		H	13
GSE41384	2012–2015	Colombia	Mix	3	3	Illumina HumanMethylation27 BeadChip		H	13
GSE36637	2012–2015	Belgium	Caucasian	4	5	Illumina HumanMethylation27 BeadChip		H	11
GSE30760	2011–2015	United Kingdom	Caucasian	15	48	Illumina HumanMethylation27 BeadChip		M	12

**Abbreviations:** CC, cervical cancer; HSIL, high-grade squamous intra-epithelial lesion; LSIL, low-grade squamous intra-epithelial lesion; B, controls with benign cervical diseases; H, healthy controls; A, autologous controls; M, mixed controls.

### Sensitivity analysis for evaluating the stable feature of pooled results

In sensitivity analyses as shown in Figure E in S1 File, sequential removal of each study produced no apparent influence upon the pooled results except one study[56].

### Publication bias of meta-analyses

In all comparisons, the shapes of funnel plots (see Figure F in S1 File) were symmetric and the values of the Egger’s test were greater than 0.05, indicating the non-existence of significant publication bias in this meta-analysis.

### Trial sequence analysis (TSA)

**Fig 9:**
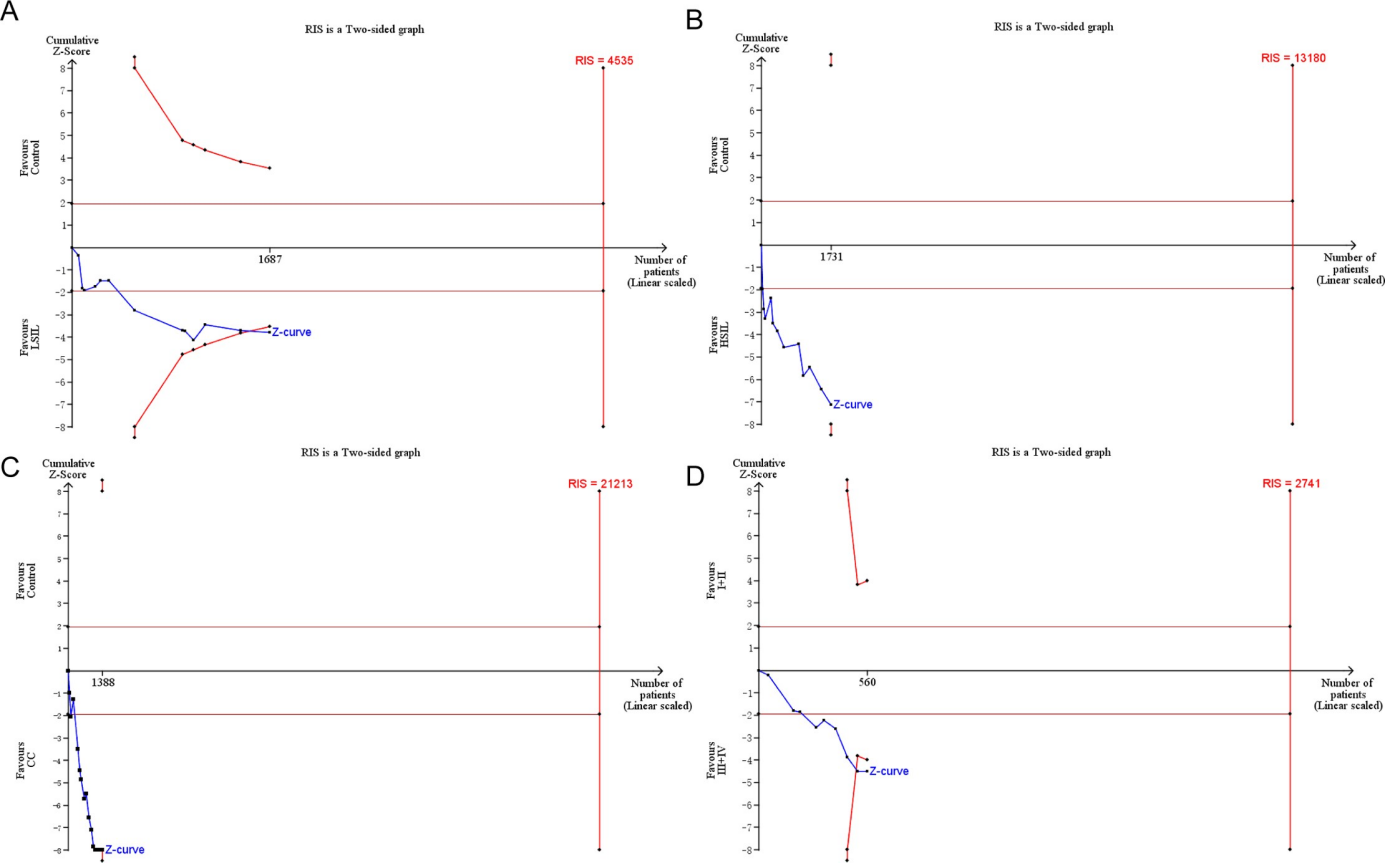
Trial sequential analysis estimating the required sample information in relation to LSIL (A), HSIL (B), CC (C) compared to controls and FIGO stage III or IV compared to FIGO stage I or II (D). **Abbreviations:** RIS, required information size.

For statistical significance, trial sequence analysis (TSA) was conducted to estimate the required information size. Based on the a priori anticipated information size method, when LSIL (the estimated required sample size of 4535 cases: Fig 9A) and cervical cancer (the estimated required sample size of 21213 cases: Fig 9C) were compared with controls, and FIGO stage III /IV were compared with FIGO stage I/II (the estimated required sample size of 2741 cases: Fig 9D), the cumulative Z-curve crossed the conventional boundary and the trial sequential monitoring boundary but not crossed required information size, which indicated the size were sufficient and significant associations were observed. However, when HSIL were compared with controls (the estimated required sample size of 13180 cases: Fig 9B), the cumulative Z-curve crossed the conventional boundary but not crossed the trial sequential monitoring boundary or required information size, which indicated that there still need more studies with large sample sizes in the future.

## Discussion

The carcinogenesis of cervical cancer involves promoter methylation or other epigenetic alterations, leading to the functional loss of TSGs[58, 59]. *MGMT* has been reported to be an important TSG that the promoter hypermethylated and silencing of *MGMT* were related to increased carcinogenic risk in several types of malignancy. Because of the inconsistent and controversial conclusions of *MGMT* promoter methylation in previous studies of cervical cancer based on different ethnicities, materials of sample and detection methods of methylation, we carried out a meta-analysis for comprehensively evaluating the association of *MGMT* promoter hypermethylation with cervical carcinogenesis.

In this meta-analysis, based upon the information of exceeding 3000 subjects from 25 relevant studies, we discovered that the methylated rates of *MGMT* progressively elevated with lesion severity, from 12.16% in control group, 20.92% in LSIL specimens, 36.33% in HSIL specimens to 41.50% in CC specimens, and that the promoter hypermethylation of *MGMT* was significant associated with the increased risk of LSIL by 1.74-fold, HSIL by 3.71-fold and CC by 7.08-fold. Besides, the results of validation by genome-wide DNA methylation array datasets extracted from GEO databases discovered that methylation of 5 loci in *MGMT* promoter CpG islands showed a great diagnostic value for the screening of cervical cancer. Such outcomes, in combination of earlier epidemiological evidence that *MGMT* promoter methylation was associated with the progression of squamous intraepithelial lesions and cervical cancer[33, 45], indicating that *MGMT* promoter hypermethylation may serve as an important biomarker for the progression of cervical carcinogenesis. Thus, the detection of the promoter methylation of *MGMT* gene could help clinicians to find out the progression of cervical carcinogenesis and even whether the patients with cervical disease is recovering or getting worse, which would improve the accuracy of diagnostic for cervical cancer.

Furthermore, whether *MGMT* aberrant promoter methylation was correlated with clinicopathological features were also analyzed based on the data presented in more than one study. It was found that *MGMT* promoter hypermethylation had significant association with the FIGO stage of cervical cancer, which was more common in advanced stage (FIGO stage III) than in low stage (FIGO stage I+II). Thus, the results further implicated that *MGMT* promoter methylation is likely to have a critical function in the progression of cervical cancer. This conclusion were consistent with previous studies in other malignant carcinoma such as follows. In *Hengstler et al*.’s research, it has been reported that *MGMT* expression is significantly associated with FIGO stages in ovarian tumors [60]. *Fu et al*. reported that *MGMT* methylation status exert a possible prognostic value in patients with duodenal adenocarcinoma in stage III [61]. Besides, in a meta-analysis by *Chen et al*., focused on the association between *MGMT* hypermethylation and non-small-cell-lung carcinoma (NSCLC), reported that *MGMT* methylation was observed to be specifically associated with NSCLC clinical stage.

Moreover, this meta-analysis was conducted to assess whether *MGMT* could be a biomarker for the prognosis of cervical cancer. Analysis from GEPIA databases showed that *MGMT* expression were associated with OS in cervical cancer, the survival percentage of patients with high *MGMT* expression is much higher than that of patients with low *MGMT* expression. We speculated that *MGMT* promoter hypermethylation could down-regulates *MGMT* mRNA expression, and progressively influenced the overall survival. This conclusion were consistent with previous studies in colorectal carcinoma, the results showed that hypermethylation of *MGMT* was an unfavourable prognostic markers in colorectal cancer[62]. However, there is still lack of related analysis between *MGMT* methylation and OS in cervical cancer. There is only one research[63] reported that *MGMT* does not seem to be implicated in OS of cervical cancer, at least not by promoter methylation-dependent mechanisms. Therefore, prospective studies could be focus on the impact of *MGMT* promoter hypermethylation on the prognosis of cervical cancer.

Moderate and extensive heterogeneity were observed in our meta-analysis for the association of *MGMT* methylation with HSIL and CC risk, respectively. Thus, such outcomes were firstly pooled by using a random-effect model, which conservatively estimates the study weights after adjusting for the inter-study variances[64]. Then, the potential sources of heterogeneity were explored by three statistical approaches, including subgroup analysis and meta-regression to identify the confounding factors associated with observed heterogeneity, and then Galbraith plots were depicted to explore the contributions of individual studies to overall heterogeneity. In the comparison between *MGMT* promoter hypermethylation and HSIL risk, the results of subgroup analysis showed that the specimen material not used by tumor tissues was probably the major origin of heterogeneity. And Galbraith plots spotted four outliers [33, 42, 56, 57] as major sources of moderate heterogeneity for the relationship between *MGMT* promoter hypermethylation and HSIL risk. Notably, these four studies collected exfoliated cells as biospecimen. In addition, the hypermethylation rates of control group in these four studies (22.81%, 26.67%, 36.90%, 50% respectively) were much higher than that of control group in overall (12.16%), suggesting the existence of inter-study differences. Besides, in the comparison between *MGMT* promoter hypermethylation and CC risk, the results of subgroup analysis and meta-regression both showed that the low quality of studies was probably the major origin of heterogeneity. Galbraith plots spotted four outliers [33, 37, 53, 56] as major sources of extensive heterogeneity, and two studies [33, 56] of them involving moderate heterogeneity for the relationship between *MGMT* promoter hypermethylation and HSIL risk. Notably, these four studies were all classified into low-quality studies, which is consistent with the conclusions of subgroup analysis and meta-regression. By appraising these four studies according to our quality scorning system, we found that the commonalities of these studies were as follows: lack of information of biospecimen characteristics, lack of blinding of laboratory staff, lack of clinical and pathological data. Besides, *MGMT* promoter hypermethylation was detected by MS-HRM in one^56^ of the two studies that regarded as both major sources of heterogeneity for the association of *MGMT* promoter hypermethylation with HSIL risk and cervical, which was the main difference in their study design from other studies.

In subgroup meta-analysis, the association between the promoter hypermethylation of *MGMT* with squamous intraepithelial lesions and cervical cancer remained significant in both Caucasian and Asian subgroups. Since no African study was available, more experiments should be performed to verify our observation in Africans together with other ethnicities in the future. Besides, in studies that regarded non-healthy as negative controls including autologous controls and controls with benign gynecological diseases, *MGMT* promoter methylation is significantly correlated with HSIL and CC risk, but not with LSIL risk, while *MGMT* methylation is significantly correlated with all SILs and cervical cancer in studies regarded healthy as controls. It could be due to the reason that the cytology of cervical cells in patients with benign gynecological disease and LSIL is similar [65].

In sensitivity analysis, sequential removal of each study had no significant impact on the pooled results except one study[56]. The methylation detection method of this study were performed by MS-HRM, which probably might be the major difference between this study and others.

However, several limitations were still needed to be mentioned in this meta-analysis. First, in most of included studies of this meta-analysis, detection of *MGMT* methylation were performed by MSP, a qualitative method relied on primer designs to guarantee the accuracy. However, different primers were designed to detect *MGMT* promoter methylation in the included studies, which may lead to the potential bias. Second, only full-text articles written in English or Chinese were included in this meta-analysis, while articles in other languages were excluded because of unreadable contents or insufficient data, thus resulting to a selection bias. Third, it is unfortunate that the pooled analyses of several clinicopathological features in this meta-analysis based on fewer than three studies, leading to an error or inaccurate conclusion in pooled results for the association between *MGMT* hypermethylation and these clinicopathological features including HPV infection, therapeutic response, age at diagnose and lymph node metastasis. Thus, more prospective clinical studies with detailed information are needed to confirm the association of *MGMT* hypermethylation with clinicopathological features in the future.

In this meta-analysis, we found that *MGMT* promoter hypermethylation was associated with squamous intra-epithelial lesion and cervical cancer. Moreover, *MGMT* promoter hypermethylation was also correlated with FIGO stage in patients with cervical cancer. Therefore, *MGMT* methylation detection might have a potential value to be an epigenetic marker for the clinical diagnosis of cervical cancer. And, it will help clinicians to decide whether to give complementary cytostatic drugs or not after the primary surgery. Besides, it also could make a contribution to explore the pathogenic mechanism of cervical cancer, as well as the preventive measures. However, prospective studies should be focus on the impact of *MGMT* methylation on the prognosis of cervical cancer.

## Supporting information

S1 File **Table A.** Preferred Reporting Items for Systematic Reviews and Meta-Analysis (PRISMA) 2009 Checklist. **Table B.** Definitions of 18 items in our quality scoring system. **Table C.** The univariate meta-regression results of the association of MGMT promoter methylation and HSIL risk. **Table D.** The univariate meta-regression results of the association of MGMT promoter methylation and CC risk. **Figure A.** Galbraith plot for the association of MGMT methylation and HSIL risk. Each number is the number of the respective study included in this meta-analysis (shown in Table 1). **Figure B.** Galbraith plot for the association of MGMT methylation and CC risk. Each number is the number of the respective study included in this meta-analysis (shown in Table 1). **Figure C.** Funnel plots for associations between MGMT promoter hypermethylation and histological type. **Figure D.** Funnel plots for associations between MGMT promoter hypermethylation and histological grade. **Figure E.** Sensitivity analyses in this meta-analysis. **Figure F.** Funnel plots in this meta-analysis.
(DOCX)

S1 Table Original minimal datasets in this meta-analysis. (XLSX)
