# Supplementary material for: Associations of MGMT promoter hypermethylation with squamous intraepithelial lesion and cervical carcinoma: A meta-analysis
Source: PLoS One. 2019 Oct 1;14(10):e0222772. doi: 10.1371/journal.pone.0222772 (PMC6772039; doi:10.1371/journal.pone.0222772)
Supplement: S1 File — Table A. Preferred Reporting Items for Systematic Reviews and Meta-Analysis (PRISMA) 2009 Checklist. Table B. Definitions of 18 items in our quality scoring system. Table C. The univariate meta-regression results of the association of MGMT promoter methylation and HSIL risk. Table D. The univariate meta-regression results of the association of MGMT promoter methylation and CC risk. Figure A. Galbraith plot for the association of MGMT methylation and HSIL risk. Each number is the number of the respective study included in this meta-analysis (shown in Table 1). Figure B. Galbraith plot for the association of MGMT methylation and CC risk. Each number is the number of the respective study included in this meta-analysis (shown in Table 1). Figure C. Funnel plots for associations between MGMT promoter hypermethylation and histological type. Figure D. Funnel plots for associations between MGMT promoter hypermethylation and histological grade. Figure E. Sensitivity analyses in this meta-analysis. Figure F. Funnel plots in this meta-analysis. (DOCX) [file pone.0222772.s001.docx]

**Supplementary Information**

**Associations of *MGMT* promoter hypermethylation with squamous intraepithelial lesion and cervical carcinoma: A meta-analysis**

Jin Huang^1^, Jia-You Luo^2^, Hong-Zhuan Tan^1*^

^1^ Department of Epidemiology and Health Statistics, School of Public Health, Central South University, Changsha, Hunan, China.

^2^ Department of Women and Children Health, School of Public Health, Central South University, Changsha, Hunan, China.

**^*^Correspondence to:** Hong-Zhuan Tan, **email:** tanhz99@qq.com

**Supplementary Table A. Preferred Reporting Items for Systematic Reviews and Meta-Analysis (PRISMA) 2009 Checklist.**

| **Section/topic** | **#** | **Checklist item** | **Reported on page #** |
| --- | --- | --- | --- |
| **TITLE** | | |  |
| Title | 1 | Identify the report as a systematic review, meta-analysis, or both. | 1 |
| **ABSTRACT** | | |  |
| Structured summary | 2 | Provide a structured summary including, as applicable: background; objectives; data sources; study eligibility criteria, participants, and interventions; study appraisal and synthesis methods; results; limitations; conclusions and implications of key findings; systematic review registration number. | 1 |
| **INTRODUCTION** | | |  |
| Rationale | 3 | Describe the rationale for the review in the context of what is already known. | 2 |
| Objectives | 4 | Provide an explicit statement of questions being addressed with reference to participants, interventions, comparisons, outcomes, and study design (PICOS). | 2 |
| **METHODS** | | |  |
| Protocol and registration | 5 | Indicate if a review protocol exists, if and where it can be accessed (e.g., Web address), and, if available, provide registration information including registration number. | 3 |
| Eligibility criteria | 6 | Specify study characteristics (e.g., PICOS, length of follow-up) and report characteristics (e.g., years considered, language, publication status) used as criteria for eligibility, giving rationale. | 3 |
| Information sources | 7 | Describe all information sources (e.g., databases with dates of coverage, contact with study authors to identify additional studies) in the search and date last searched. | 3 |
| Search | 8 | Present full electronic search strategy for at least one database, including any limits used, such that it could be repeated. | 3 |
| **Section/topic** | **#** | **Checklist item** | **Reported on page #** |
| Study selection | 9 | State the process for selecting studies (i.e., screening, eligibility, included in systematic review, and, if applicable, included in the meta-analysis). | 3 |
| Data collection process | 10 | Describe method of data extraction from reports (e.g., piloted forms, independently, in duplicate) and any processes for obtaining and confirming data from investigators. | 3 |
| Data items | 11 | List and define all variables for which data were sought (e.g., PICOS, funding sources) and any assumptions and simplifications made. | 3 |
| Risk of bias in individual studies | 12 | Describe methods used for assessing risk of bias of individual studies (including specification of whether this was done at the study or outcome level), and how this information is to be used in any data synthesis. | 6 |
| Summary measures | 13 | State the principal summary measures (e.g., risk ratio, difference in means). | 3&4 |
| Synthesis of results | 14 | Describe the methods of handling data and combining results of studies, if done, including measures of consistency (e.g., I^2^) for each meta-analysis. | 4 |

| Risk of bias across studies | 15 | Specify any assessment of risk of bias that may affect the cumulative evidence (e.g., publication bias, selective reporting within studies). | 6 |
| --- | --- | --- | --- |
| Additional analyses | 16 | Describe methods of additional analyses (e.g., sensitivity or subgroup analyses, meta-regression), if done, indicating which were pre-specified. | 4 |
| **RESULTS** | | |  |
| Study selection | 17 | Give numbers of studies screened, assessed for eligibility, and included in the review, with reasons for exclusions at each stage, ideally with a flow diagram. | 4 |
| Study characteristics | 18 | For each study, present characteristics for which data were extracted (e.g., study size, PICOS, follow-up period) and provide the citations. | 4 |
| Risk of bias within studies | 19 | Present data on risk of bias of each study and, if available, any outcome level assessment (see item 12). | 6 |
| **Section/topic** | **#** | **Checklist item** | **Reported on page #** |
| Results of individual studies | 20 | For all outcomes considered (benefits or harms), present, for each study: (a) simple summary data for each intervention group (b) effect estimates and confidence intervals, ideally with a forest plot. | 5 |
| Synthesis of results | 21 | Present results of each meta-analysis done, including confidence intervals and measures of consistency. | 5 |
| Risk of bias across studies | 22 | Present results of any assessment of risk of bias across studies (see Item 15). | 6 |
| Additional analysis | 23 | Give results of additional analyses, if done (e.g., sensitivity or subgroup analyses, meta-regression [see Item 16]). | 5&6 |
| **DISCUSSION** | | |  |
| Summary of evidence | 24 | Summarize the main findings including the strength of evidence for each main outcome; consider their relevance to key groups (e.g., healthcare providers, users, and policy makers). | 6&7 |
| Limitations | 25 | Discuss limitations at study and outcome level (e.g., risk of bias), and at review-level (e.g., incomplete retrieval of identified research, reporting bias). | 8 |
| Conclusions | 26 | Provide a general interpretation of the results in the context of other evidence, and implications for future research. | 8 |
| **FUNDING** | | |  |
| Funding | 27 | Describe sources of funding for the systematic review and other support (e.g., supply of data); role of funders for the systematic review. | 9 |

*From:*  Moher D, Liberati A, Tetzlaff J, Altman DG, The PRISMA Group (2009). Preferred Reporting Items for Systematic Reviews and Meta-Analyses: The PRISMA Statement. PLoS Med 6(7): e1000097. doi:10.1371/journal.pmed1000097

**Supplementary Table B: Definitions of 18 items in our quality scoring system.**

| **Study design** |
| --- |
| 1.Study objective definition: state the study objectives, prespecified hypothesis or study protocol  2. Sample size: state a statistical sample size or power calculation  3. Population source: state health care setting from which patients were recruited  4. Population selection criteria: state inclusion or exclusion criteria  5. Population demographic characteristics: state the population demographic characteristics (e.g., age, age ate primiparity and menopausal status)  6. Diagnosis of patients: state the criteria or guidelines to diagnose the included patients |
| **Biospecimen information** |
| 1. Biospecimen characteristics: state biospecimen type and anatmical site  2. Biospecimen management: state the methods of collection and storage |
| **Methylation detection** |
| 1. Assay method: state the type of assay method used to detect methylation status (MSP, BSP or pyrosequencing, etc)  2. Primer designs: state the primer sequences  3. Quality control: state the method of quality control  4. Blinding of laboratory staff: methylation detection done under "blinded" condition |
| **Clinicopathological features** |
| 1. Clinical and pathological data: state the clinical and pathological data (such as tumor type, stage and grade)  2. Conventional risk factors: state the conventional risk factors (such as HPV infection, smoking habit)  3. Other biomarkers: state other biomarker relating with disease (such as methylation status of other genes, point mutation and expression level) |
| **Results analysis** |
| 1. Univariate estimate: report the effect of methylation status on outcome  2. Multivariate estimate: adjusted for risk factors or other biomarkers  3. Missing data: state the number of patients with missing data and how to deal with it |

**Supplement Table C.** The univariate meta-regression results of the association of *MGMT* promoter methylation and HSIL risk.

| Covariates | Coefficient | Standard error | *P* value | 95% CI | τ^2^ value (%) | I^2^ res(%) |
| --- | --- | --- | --- | --- | --- | --- |
| Ethnicity | 0.022 | 0.786 | 0.978 | -1.709~1.753 | -22.07 | 69.44 |
| Source of controls | -0.001 | 0.741 | 0.999 | -1.632~1.630 | -21.33 | 69.47 |
| Materials | -0.612 | 0.906 | 0.513 | -2.607~1.382 | -10.56 | 68.61 |
| Publication year | 0.773 | 0.775 | 0.340 | -0.933~2.479 | -15.72 | 69.45 |
| Quality of studies | 0.243 | 0.750 | 0.752 | -1.408~1.895 | -18.92 | 69.38 |

**Supplement Table D.** The univariate meta-regression results of the association of *MGMT* promoter methylation and CC risk.

| Covariates | Coefficient | Standard error | *P* value | 95% CI | τ^2^ value (%) | I^2^ res(%) |
| --- | --- | --- | --- | --- | --- | --- |
| Ethnicity | -1.207 | 0.984 | 0.248 | -3.400~0.985 | -3.34 | 77.06 |
| Source of controls | 0.113 | 0.726 | 0.880 | -1.504~1.730 | -7.07 | 75.48 |
| Materials | 0.335 | 0.725 | 0.654 | -1.280~1.951 | -8.65 | 76.94 |
| Publication year | 0.322 | 0.548 | 0.570 | -0.899~1.543 | -10.32 | 77.08 |
| Quality of studies | -2.933 | 0.518 | **<0.001** | -4.086~-1.780 | 0.00 | 100.00 |

**Supplementary Figure A.** Galbraith plot for the association of *MGMT* methylation and HSIL risk. Each number is the number of the respective study included in this meta-analysis (shown in Table 1).

**Supplementary Figure B.** Galbraith plot for the association of *MGMT* methylation and CC risk. Each number is the number of the respective study included in this meta-analysis (shown in Table 1).


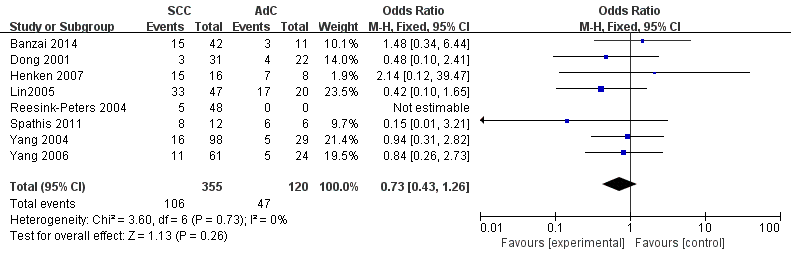


**Supplementary Figure C.** Funnel plots for associations between *MGMT* promoter hypermethylation and histological type. The squares represent the ORs for individual studies. The size of the square reflects the weight of included studies. Bars represent the 95% confidence intervals (CIs). The center of the diamond represents the summary effect size.

**Abbreviations:** SCC, squamous cell carcinoma; AdC, adenocinoma.


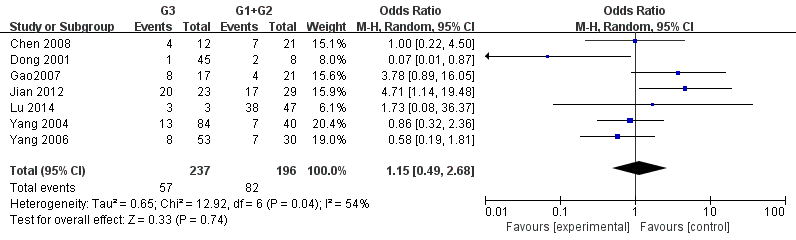


**Supplementary Figure D.** Funnel plots for associations between *MGMT* promoter hypermethylation and histological grade. The squares represent the ORs for individual studies. The size of the square reflects the weight of included studies. Bars represent the 95% confidence intervals (CIs). The center of the diamond represents the summary effect size.

**Abbreviations:** G1, histological grade 1; G2+3, histological grade 2 and grade3.


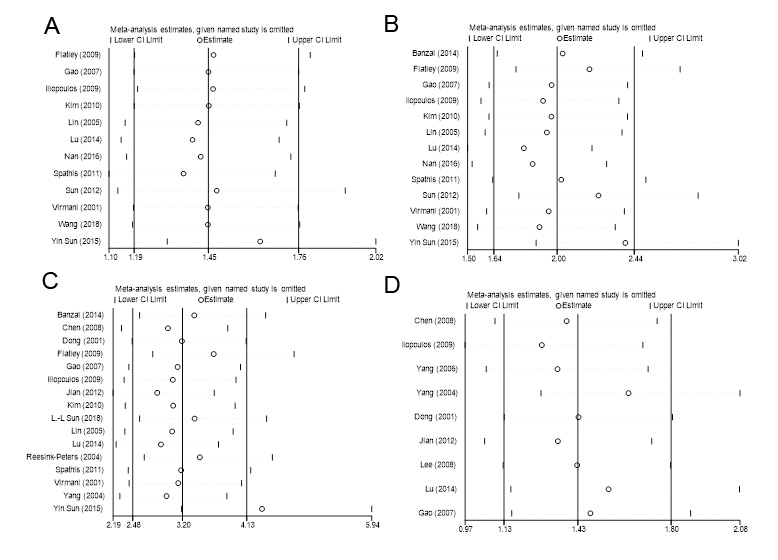


**Supplementary Figure E. Sensitivity analyses in this meta-analysis.** (A) sensitivity analyses for the association between *MGMT* hypermethylation and LSIL risk; (B) sensitivity analyses for the association between *MGMT* promoter hypermethylation and HSIL risk; (C) sensitivity analyses for the association between *MGMT* promoter hypermethylation and CC risk; (D) sensitivity analyses for the association between *MGMT* promoter hypermethylation and FIGO stage. The results were computed by sequentially omitting each study. Bar represents 95% CI. The center of bars represents the summary effects when omitting corresponding studies.


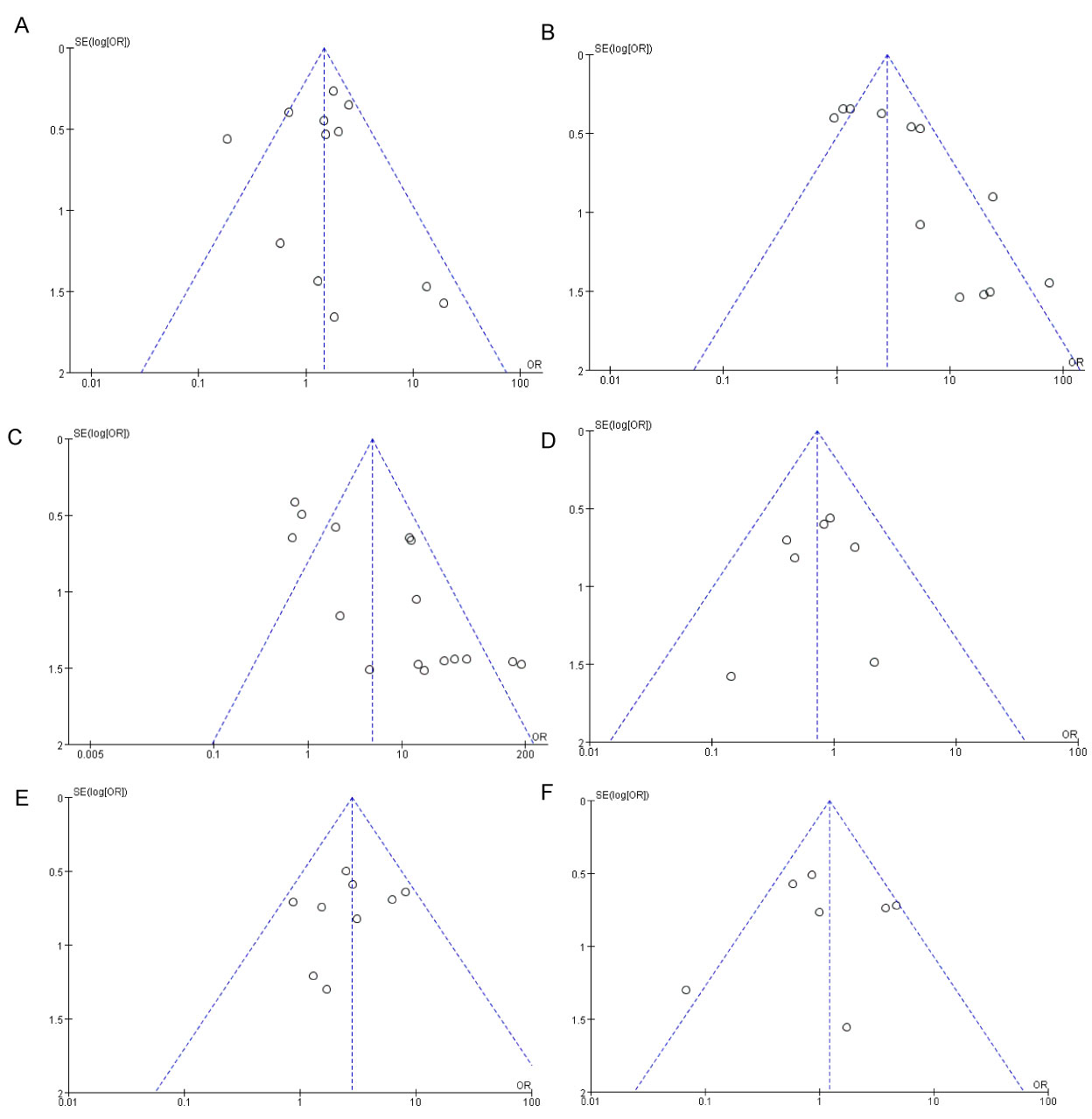


**Supplementary Figure F. Funnel plots in this meta-analysis.** (A) funnel plot for the association between *MGMT* promoter hypermethylation and LSIL risk; (B) funnel plot for the association between *MGMT* promoter hypermethylation and HSIL risk; (C) funnel plot for the association between *MGMT* promoter hypermethylation and CC risk; (D) funnel plot for the association between *MGMT* promoter hypermethylation and histological type; (E) funnel plot for the association between *MGMT* promoter hypermethylation and FIGO stage; (F) funnel plot for the association between *MGMT* promoter hypermethylation and histological grade.
